# Variation in the use of primary care-led investigations prior to a cancer diagnosis: analysis of the National Cancer Diagnosis Audit

**DOI:** 10.1136/bmjqs-2024-017264

**Published:** 2024-10-23

**Authors:** Nurunnahar Akter, Georgios Lyratzopoulos, Ruth Swann, Greg Rubin, Sean McPhail, Meena Rafiq, Abodunrin Aminu, Nadine Zakkak, Gary Abel

**Affiliations:** 1 Department of Health Data Science, Institute of Population Health, University of Liverpool, Liverpool, UK; 2 Department of Health & Community Sciences, University of Exeter Medical School, Exeter, UK; 3 Epidemiology of Cancer and Healthcare Outcomes (ECHO) Group, University College London, London, UK; 4 Cancer Research UK, London, UK; 5 NHS England, London, UK; 6 Newcastle University, Newcastle upon Tyne, UK; 7 NHS England North, Leeds, UK; 8 Department of General Practice and Primary Care, University of Melbourne, Melbourne, Victoria, Australia; 9 Research Department of Epidemiology and Public Health, University College London, London, UK

**Keywords:** diagnostic imaging, primary care, health services research, statistics

## Abstract

**Abstract:**

**Introduction:**

Use of investigations can help support the diagnostic process of patients with cancer in primary care, but the size of variation between patient group and between practices is unclear.

**Methods:**

We analysed data on 53 252 patients from 1868 general practices included in the National Cancer Diagnosis Audit 2018 using a sequence of logistic regression models to quantify and explain practice-level variation in investigation use, accounting for patient-level case-mix and practice characteristics. Four types of investigations were considered: any investigation, blood tests, imaging and endoscopy.

**Results:**

Large variation in practice use was observed (OR for 97.5th to 2.5th centile being 4.02, 4.33 and 3.12, respectively for any investigation, blood test and imaging). After accounting for patient case-mix, the spread of practice variation increased further to 5.61, 6.30 and 3.60 denoting that patients with characteristics associated with higher use (ie, certain cancer sites) are over-represented among practices with lower than the national average use of such investigation. Practice characteristics explained very little of observed variation, except for rurality (rural practices having lower use of any investigation) and concentration of older age patients (practices with older patients being more likely to use all types of investigations).

**Conclusion:**

There is very large variation between practices in use of investigation in patients with cancer as part of the diagnostic process. It is conceivable that the diagnostic process can be improved if investigation use was to be increased in lower use practices, although it is also possible that there is overtesting in practices with very high use of investigations, and in fact both undertesting and overtesting may co-exist.

**WHAT IS ALREADY KNOWN ON THIS TOPIC**

**WHAT THIS STUDY ADDS**

- This study identified substantial variation in the use of investigations among practices in primary care for patients with cancer, which could not be attributed to particular practice or patient characteristic.

**HOW THIS STUDY MIGHT AFFECT RESEARCH, PRACTICE OR POLICY**

- The finding that substantial variation in the use of investigations between primary care providers cannot be attributed to the characteristics of general practices (such as size, rurality, quality of care and the age of registered patients) implies that this variability reflects an embedded tendency rather than any organisational or structural issues.
- It is unclear from our data whether this variation should be reduced.
- Based on this study, any interventions to reduce this variation should focus on the rate of investigation use itself, rather than particular types of practices.

## Introduction

 One in every two people will develop some form of cancer over their lifetime.^1^ Timely diagnosis of cancer increases the chance of a better outcome of treatment and prolonged survival. Delays in diagnosis are a cause for concern for policymakers, professionals and the public. In England, primary care is the first place of contact for most patients who are experiencing symptoms. There is growing evidence of an association between the length of diagnostic intervals (from first clinical presentation to diagnosis) and clinical outcome and patient experience,^2^ alongside evidence of significant practice variation in primary care diagnostic activity prediagnosis.^3^,^5^ Prior work has examined the use of common blood test by sociodemographic factors, and presenting symptom, and the impact of primary care tests on diagnostic intervals.^6^,^8^ Although there were great improvements over time in the diagnostic process of patients who present to general practice with symptoms of a subsequently diagnosed cancer in England,^9^ little is known about the role of tests and related variation by patients and practice-level factors.

Previous studies have examined practice-level variation in investigations that are used as part of the cancer diagnostic workup, and the association of that variation with practice characteristics.^5 10 11^ However, these studies considered overall use of these investigations in patients registered with the practice (including both patients with cancer and, chiefly, patients without cancer). Given these previous findings, along with those from other clinical areas that show substantial variation among individual clinicians and providers,^12^,^15^ it is important to examine the variation in primary care-led investigations (including blood tests, endoscopy and imaging, both collectively and individually) used as part of the diagnostic assessment in patients subsequently diagnosed with cancer, as well as how this variation is influenced by various patient and general practice factors. At the outset, we aimed to quantify practice variation and identify any likely contributing factors, primarily related to patient case-mix and practice characteristics.

## Method

### Study design

**Figure 1:**
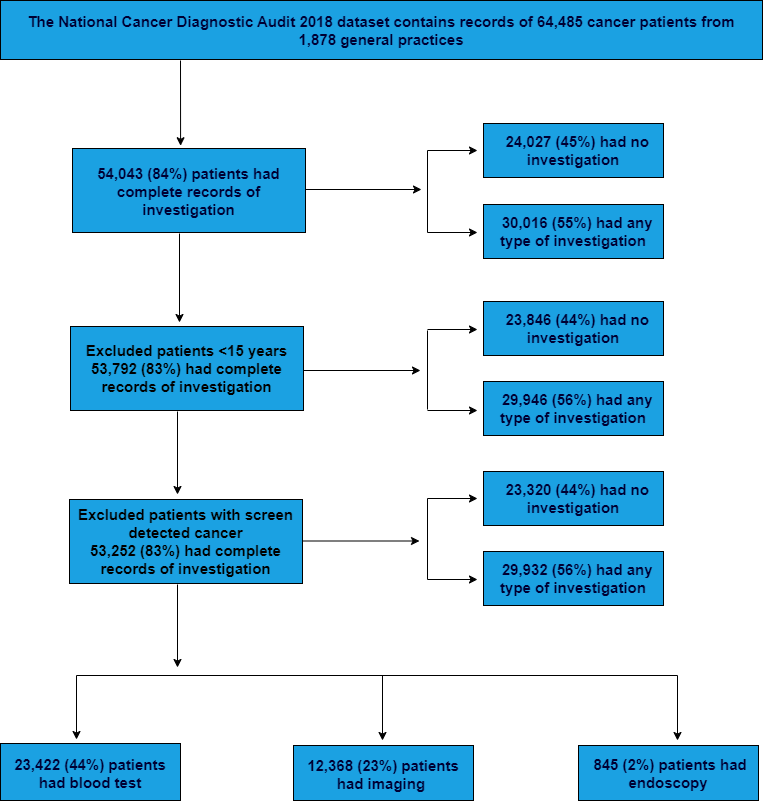
Flow diagram of the selection of the analysis sample. Note that a patient could have had more than one of the three test categories examined. Some patients may be represented more than once if they had more than one tumour diagnosed in 2018.

In this paper, we examine the tests ordered by general practitioners before referring patients to a specialist. The National Cancer Diagnosis Audit (NCDA) 2018 dataset was used to investigate the use of various types of tests in primary care patients who were subsequently diagnosed with cancer. The information on the investigation process and other characteristics for patients who were diagnosed with cancer in 2018 was collected by participating general practices based on reviewing information in the primary care records. Included patients were identified by the English Cancer Registry (National Disease Registration Service). In situ tumours and non-melanoma skin cancer were excluded from the audit. Those completing the audit used their judgement as to what investigations were relevant to a cancer diagnosis. The details about data collection have been described in previous publications.^16 17^ The patients in the audit were representative of the national cohort of patients with cancer in terms of age, gender, stage of cancer and cancer site, and the characteristics of the participating practices were comparable to non-participating practices.^17^ This study included the information of 53 252 patients aged 15 years or over at diagnosis with non-screen detected cancer from 1868 practices and who have complete information on investigation status. The derivation of the analysis sample is shown in figure 1.

### Outcome variables

The audit questionnaire collected information about various types of investigation used in primary care patients prior to a referral to specialist or emergency presentation that results in a diagnosis. In this study, four binary-coded variables were defined based on whether a patient had one or more of the following categories of tests: blood test, imaging, endoscopy or any investigation (see box 1 for details).

Box 1 **Test-related questionnaires and response options used for NCDA data collection**

Q1. Were any investigations ordered prior to specialist referral?

- Yes
- No
- Not known

Q2. If yes, please select which investigation(s) were ordered?*

- Blood test—amylase
- Blood test—cancer biomarker (PSA, CEA, CA125, CA19.9, other)
- Blood test—FBC
- Blood test—inflammatory marker (ESR, CRP)
- Blood test—liver function tests
- Blood test—serum proteins/paraproteins
- Blood test—U&E, eGFR
- Blood test—other
- Bone profile
- Bronchoscopy
- Colonoscopy
- Colposcopy
- Contrast radiology—other
- Contrast radiology (barium swallow, meal, enema/other)
- CT abdomen
- CT brain
- CT chest
- CT other
- Cytology
- Endoscopy upper GI
- Ferritin
- Faecal immunochemical test
- Flexible cystoscopy
- Flexible Sigmoidoscopy
- MRI brain
- MRI spine
- MRI other
- Ultrasound other
- Ultrasound abdomen
- Ultrasound neck
- Ultrasound pelvic
- Ultrasound transvaginal
- X-ray chest
- X-ray skeletal
- X-ray other
- Other
- Not known
- Not applicable

*Our study classified investigations as follows: any investigation (if patients had at least one of the investigations carried out from the list), blood test (ferritin, bone profile and all categories of blood tests), imaging (all types of ultrasound, CT, MRI, contrast radiology and X-ray), endoscopy (upper GI, colonoscopy, flexible cystoscopy/sigmoidoscopy, bronchoscopy and colposcopy). Not known and not applicable investigations were excluded from the analysis. Some patients may have more than one investigation.CA, cancer antigen; CEA, carcinoembryonic antigen; CRP, C reactive protein; eGFR: estimated glomerular filtration rate; ESR, erythrocyte sedimentation rate; FBC, full blood count; GI, gastrointestinal; NCDA, National Cancer Diagnosis Audit; PSA, prostate-specific antigen; U&E, urea and electrolytes.

### Exposure variables

Patient-level exposure variables included gender (male, female), age group (15–49, 50–59, 60–69, 70–79, 80+ years), ethnicity (white, non-white), deprivation quintile (based on the 2019 index of multiple deprivation of lower layer super output area of English residence), count of comorbidities (0, 1, 2, 3 and ≥4) and 29 cancer sites based on the 10th revision of the International Classification of Diseases code (see online supplemental appendix table 1).

A pseudonymised practice identifier was available along with the following practice-level variables: practice-level deprivation quintile, rurality (urban, rural), practice list size (size 1: <6000 patients, size 2: 6000–12000, size 3: >12 000), indirectly age-gender standardised two-teek wait (TWW) referral ratio, patients per practice, maximum quality and outcome framework score, patients aged over 65+ years per practice and variables representing key aspects of patients’ experience (access, continuity, satisfaction and doctor communication) as reported by the General Practice Patient Survey.^1018^,^20^

### Statistical analysis

Separately for each of the three investigation types (any test, blood test and imaging) treated as an outcome, four logistic regression models were employed. Three of these models employ a random effect for practice, which can be used to quantify variation in investigation use between practices after accounting for the role of chance that can inflate the raw observed variation.^4^ The four models were:

- Model 1 included random effect for general practice without any fixed effect variables; this model simply characterises the overall variation in investigation use between practices, after accounting for chance and without examining any other factors.
- Model 2 included all patient-level exposure variables listed above as fixed effects but without a random effect for practice; this model examines associations between person-level factors and use of tests across the entire sample, without taking into account clustering of observations within practices or practice characteristics.
- Model 3 included all variables considered in both models 1 and 2, that is, all patient-level variables and a random effect for general practice; this model examines the contribution of each patient-level variable additionally accounting for confounding by patients clustering within general practices, and vice versa (ie, it examines general practice variation after accounting for confounding patient-level variables).
- Model 4 was identical to model 3 but with the addition of fixed effect variables for practice characteristics; this model examines the possible role of different practice characteristics above and beyond their patient-level case-mix and the possible clustering of patients with different characteristics in different practices.

**Figure 2:**
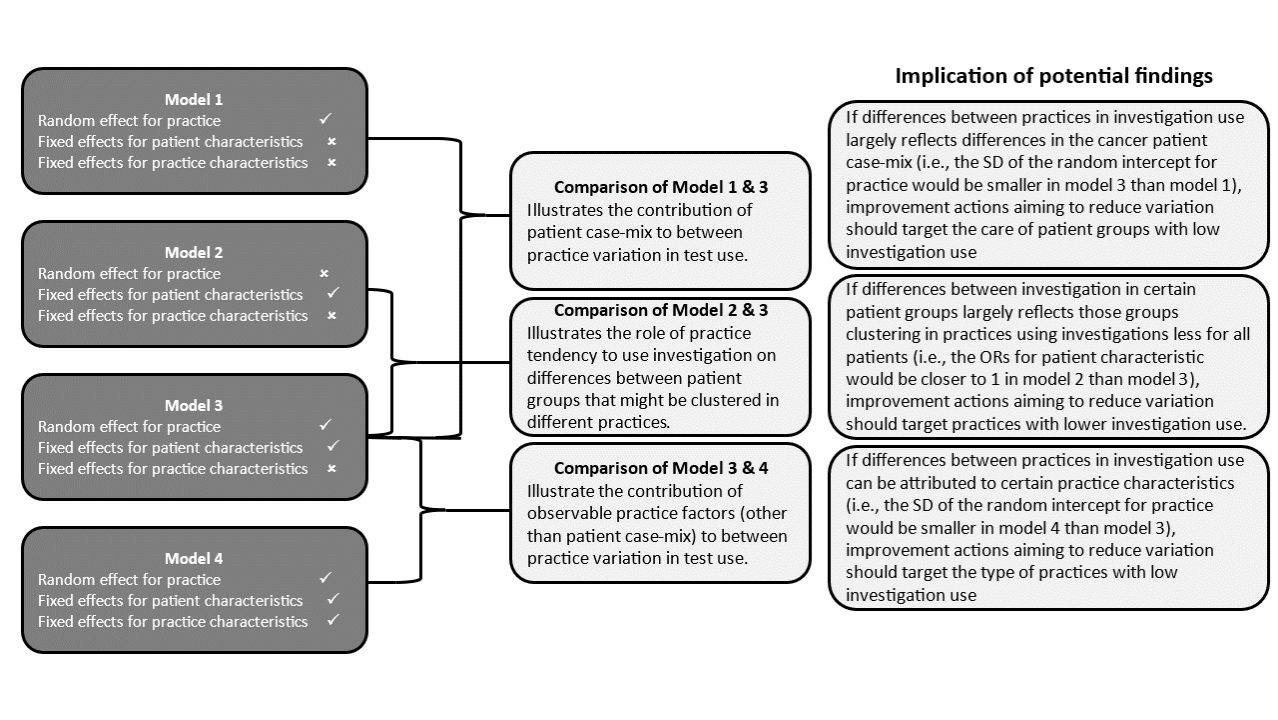
Modelling strategy and informative comparisons of model outputs, and their interpretation.

Building on the outputs of the four models, the following informative comparisons were performed (see figure 2):

- Comparison of model 1 (table 1) and model 3 (table 2) outputs illustrates the extent to which patient case-mix contributes to between-practice variation in test use.
- Comparison of model 2 (online supplemental appendix table 2) and model 3 outputs illustrates the degree to which variation in test use between different patient groups relates to their potential clustering in practices with higher/lower than average use of investigation.
- Comparison between model 3 and model 4 (online supplemental appendix table 3) outputs demonstrates the role of structural or functional practice characteristics, once patient-level differences and patient clustering into different practices is taken into account. To facilitate comparisons of the effect sizes, continuous variables were standardised before fitting the model so that the ORs estimated from the regression models correspond to 1 SD change in the exposure variables. All statistical analyses were conducted in Stata SE V.17 (StataCorp).

**Table 1:** Between-practice variation in the unadjusted and adjusted mixed model by type of investigation

Model	Any investigationSD (95% CI)	Blood testSD (95% CI)	ImagingSD (95% CI)
Model 1* (unadjusted)	0.36 (0.33 to 0.38)	0.37 (0.35 to 0.40)	0.29 (0.26 to 0.33)
Model 3* (adjusted for patient factors)	0.44 (0.41 to 0.47)	0.47 (0.44 to 0.51)	0.32 (0.29 to 0.36)
Model 4[Table-fn T1_FN1] (adjusted for patients and practice factors)	0.44 (0.41 to 0.48)	0.47 (0.43 to 0.50)	0.32 (0.29 to 0.36)

* P<0.001 for all models.

**Table 2:** ORs and 95% CIs in the adjusted mixed model of investigation use among patients subsequently diagnosed with cancer (model 3)

	Any investigation			Blood tests			Imaging		
	OR (95% CI)	P value	Globalp Value	OR (95% CI)	P value	Globalp Value	OR (95% CI)	P value	Globalp Value
Gender									
Male	Ref								
Female	1.03 (0.98, 1.08)	0.244		0.98 (0.94, 1.03)	0.539		1.08 (1.02, 1.14)	0.008	
Ethnicity									
White	Ref								
Non-white	0.98 (0.91, 1.06)	0.631		0.95 (0.88, 1.03)	0.194		0.99 (0.91, 1.08)	0.823	
Age group (years)									
15–49	0.94 (0.86, 1.03)	0.181	<0.001	0.87 (0.78, 0.96)	0.005	0.022	1.30 (1.18, 1.43)	<0.001	<0.001
50–59	0.97 (0.90, 1.04)	0.383		0.93 (0.87, 1.00)	0.066		1.03 (0.95, 1.12)	0.434	
60–69	Ref								
70–79	0.96 (0.90, 1.01)	0.143		1.00 (0.95, 1.06)	0.899		0.96 (0.90, 1.03)	0.239	
80+	0.85 (0.80, 0.91)	<0.001		0.97 (0.91, 1.03)	0.325		0.78 (0.73, 0.84)	<0.001	
Deprivation									
1—least deprived	Ref								
2	1.00 (0.93, 1.02)	0.993	0.304	1.03 (0.96, 1.01)	0.419	0.002	0.98 (0.91, 1.07)	0.716	0.449
3	0.95 (0.89, 1.02)	0.175		0.92 (0.86, 1.12)	0.025		0.97 (0.89, 1.05)	0.391	
4	0.97 (0.91, 1.05)	0.502		0.93 (0.87, 1.00)	0.064		1.04 (0.96, 1.12)	0.365	
5—most deprived	0.94 (0.87, 1.01)	0.088		0.91 (0.84, 0.98)	0.011		0.99 (0.92, 1.07)	0.856	
Morbidities									
0	Ref								
1	0.85 (0.80, 0.91)	<0.001	<0.001	0.86 (0.81, 0.92)	<0.001	<0.001	0.92 (0.85, 0.98)	0.012	<0.001
2	0.77 (0.72, 0.83)	<0.001		0.81 (0.75, 0.86)	<0.001		0.85 (0.79, 0.92)	<0.001	
3	0.72 (0.66, 0.77)	<0.001		0.76 (0.70, 0.82)	<0.001		0.79 (0.73, 0.86)	<0.001	
4+	0.63 (0.58, 0.68)	<0.001		0.71 (0.65, 0.77)	<0.001		0.71 (0.64, 0.78)	<0.001	
Cancer site									
Bladder	1.04 (0.91, 1.19)	0.554	<0.001	0.44 (0.38, 0.50)	<0.001	<0.001	0.84 (0.71, 0.99)	0.045	<0.001
Brain	0.20 (0.16, 0.24)	<0.001		0.18 (0.14, 0.22)	<0.001		0.60 (0.47, 0.78)	<0.001	
Breast	0.03 (0.02, 0.03)	<0.001		0.02 (0.02, 0.03)	<0.001		0.13 (0.11, 0.16)	<0.001	
Cervical	0.42 (0.33, 0.54)	<0.001		0.17 (0.13, 0.23)	<0.001		0.95 (0.68, 1.33)	0.775	
Colon	Ref								
Endometrial	0.43 (0.38, 0.49)	0.489		0.21 (0.18, 0.24)	<0.001		1.87 (1.62, 2.17)	<0.001	
Gallbladder	1.13 (0.79, 1.62)	<0.001		0.88 (0.62, 1.23)	0.445		3.35 (2.38, 4.72)	<0.001	
Laryngeal	0.36 (0.28, 0.45)	<0.001		0.16 (0.12, 0.21)	<0.001		2.17 (1.69, 2.78)	<0.001	
Leukaemia	1.50 (1.29, 1.74)	0.029		1.70 (1.46, 1.96)	<0.001		0.72 (0.60, 0.87)	0.001	
Liver	0.83 (0.71, 0.98)	0.371		0.72 (0.61, 0.84)	<0.001		2.78 (2.34, 3.31)	0.005	
Lung	0.96 (0.88, 1.05)	0.015		0.30 (0.28, 0.33)	<0.001		6.31 (5.70, 6.98)	<0.001	
Lymphoma	0.87 (0.77, 0.97)	<0.001		0.60 (0.53, 0.67)	<0.001		2.54 (2.24, 2.88)	<0.001	
Melanoma	0.04 (0.04, 0.05)	0.003		0.02 (0.01, 0.02)	<0.001		0.08 (0.06, 0.11)	<0.001	
Mesothelioma	1.42 (1.13, 1.79)	<0.001		0.37 (0.30, 0.46)	<0.001		10.61 (8.48, 13.27)	<0.001	
Myeloma	1.51 (1.27, 1.80)	<0.001		1.45 (1.22, 1.71)	<0.001		2.03 (1.70, 2.42)	0.002	
Oesophageal	0.56 (0.49, 0.64)	<0.001		0.46 (0.40, 0.52)	<0.001		0.66 (0.55, 0.80)	<0.001	
Oral	0.18 (0.15, 0.20)	0.035		0.14 (0.12, 0.16)	<0.001		0.81 (0.68, 0.98)	0.035	
Ovarian	1.18 (1.01, 1.38)	0.003		0.84 (0.73, 0.97)	0.021		4.00 (3.43, 4.66)	<0.001	
Pancreatic	1.22 (1.07, 1.39)	<0.001		1.03 (0.91, 1.17)	0.602		2.54 (2.10, 2.91)	<0.001	
Prostate	3.36 (3.04, 3.70)	<0.001		3.47 (3.16, 3.81)	<0.001		0.45 (0.40, 0.50)	<0.001	
Rectal	0.74 (0.66, 0.84)	<0.001		0.78 (0.69, 0.88)	<0.001		0.34 (0.27, 0.41)	<0.001	
Renal	0.73 (0.65, 0.83)	0.821		0.37 (0.32, 0.42)	<0.001		2.59 (2.26, 2.97)	<0.001	
Small intestine	0.97 (0.73, 1.28)	0.172		0.83 (0.63, 1.09)	0.188		1.37 (1.00, 1.88)	0.049	
Stomach	1.12 (0.95, 1.31)	0.033		0.91 (0.78, 1.06)	0.246		1.02 (0.84, 1.23)	0.826	
Testicular	0.77 (0.61, 0.98)	0.527		0.07 (0.05, 0.10)	<0.001		4.84 (3.83, 6.13)	<0.001	
Thyroid	1.07 (0.87, 1.30)	<0.001		0.47 (0.39, 0.57)	<0.001		4.26 (3.52, 5.16)	<0.001	
Vulval	0.09 (0.06, 0.14)	<0.001		0.05 (0.03, 0.09)	<0.001		0.26 (0.14, 0.50)	<0.001	
Unknown primary	0.77 (0.67, 0.90)	<0.001		0.59 (0.51, 0.68)	<0.001		2.51 (2.14, 2.95)	<0.001	
Other	0.45 (0.40, 0.50)	0.489		0.31 (0.27, 0.34)	<0.001		1.47 (1.28, 1.67)	<0.001	

Ref, reference.

## Results

### Description of sample

53 252 patients from 1868 general practices who were subsequently diagnosed with cancer had complete information on the usage of investigation. The median number of patients with cancer per practice was 23 (IQR 11–40). There were more males in the sample than females (55% vs 45%) (see online supplemental appendix table 4). The majority of the patients (91%) were white. Nearly 77% of patients had at least one chronic disease, whereas 23% had three or more. Almost three-quarters of patients were 60 years or older at diagnosis. As previously reported, the patient population included in the NCDA was representative of those diagnosed with cancer in England with regard to age, gender, deprivation, ethnicity and cancer site.^16^

### Use of investigations

Overall, 56% (29 932/53 252) of patients in the analysis sample underwent at least one investigation before being diagnosed with cancer, but this percentage varied between practices (IQR of practice-level percentage 47%–68%). There was also substantial variation by cancer type and patient demographics (see online supplemental appendix table 4). When considering specific types of investigations, 44% (23 422/53 252) had blood test, 23% (12 368/53 252) underwent imaging and only 2% (845/53 252) had endoscopies. The use of these types of tests varied between practices with the practice-level IQRs being 33%–53%, 15%–31% and 0%–2%, respectively.

### Results from models

Due to the low number of endoscopies in the dataset, it was difficult to achieve convergence of all models for this outcome; therefore, only results for any investigation, blood test and imaging use are reported. In model 1 (including random intercept for general practice only to quantify practice variation after accounting for chance), the estimated SD of between-practice use on the log-odds scale was 0.36 (95% CI 0.33 to 0.38) for any investigation, 0.37 (0.35 to 0.40) for blood tests and 0.29 (0.26 to 0.33) for imaging, respectively. These estimates translate to ORs of 4.02, 4.33 and 3.12, respectively covering 95% midrange (ie, the 2.5th to 97.5th centiles of practice distribution). This means that there was greater than fourfold variation for use of any investigation and blood tests, and over a threefold variation in imaging, between the practices that use them the most and those that use them the least (excluding 2.5% of practices at each extreme).^4^

Considering the outputs of model 3 that included both a practice random effect and patient-level variables, the estimated SD of between-practice use increased to 0.44 (95% CI 0.41 to 0.47, p<0.001) for any test, 0.47 (0.44 to 0.51, p<0.001) for blood tests and 0.32 (0.29 to 0.36, p<0.001) for imaging. The corresponding ORs covering the 95% mid-range of practices were 5.61, 6.30 and 3.60, respectively. This means that patient case-mix was hiding variation between practices, suggesting that those with a higher proportion of patients in groups where tests were used most often were generally less likely to use tests across all their patients, and vice versa. Additional analyses showed that the increased between-practice variation in the adjusted model (model 3) was mostly driven by cancer site, meaning that patients with cancers which were overall associated with higher use of tests were more likely to belong to practices with lower than average use of investigation for any cancer site (online supplemental appendix table 5).

Considering the outputs of model 2 that examined patient-level factors without adjustment for practice variation, the odds of any investigation and imaging was lower in the oldest age group (80+ years) compared with the 60–69 years age group (see online supplemental appendix table 2). Female patients had slightly higher imaging than males (OR 1.08, 95% CI 1.02 to 1.14, p=0.007). Compared with white, non-white patients had fewer blood tests (OR 0.91, 95% CI 0.85 to 0.98, p=0.012). There was no significant association between the level of deprivation and the use of any type of investigation except blood tests where the most deprived group had fewer blood tests than the least deprived. There was decreased use of all types of investigations with increased comorbidities. Including the random effect for general practice in the case-mix (model 3) had no impact on person-level investigations; however, variation in the use of blood tests became non-significant for ethnicity (table 2).

The inclusion of practice factors in model 4 resulted in a very small change in the odds associated with patient factors for all types of investigations, with no material change in the SD of between-practice variation compared with model 3. However, we found (see online supplemental appendix table 3) that patients registered at rural practices had lower odds of undergoing any investigation (OR 0.86, 95% CI 0.76 to 0.96, p=0.010) compared with urban practices. Additionally, patients registered at practices with a higher proportion of patients aged 65 years or older had significantly higher odds of undergoing all kinds of investigations. Furthermore, patients registered at practices with larger list sizes had higher odds of blood test use (size 2: OR 1.11, 95% CI 1.02 to 1.21; size 3: OR 1.14, 95% CI 1.03 to 1.27; global p=0.024) but no such association was observed for other investigations.

Finally, patients registered at practices with higher TWW referral ratio had higher odds of imaging. There was no evidence that practice-level deprivation, patients per practice and variables representing key aspects of patients’ experience (access, continuity, satisfaction and doctor communication) had any impact on practice test use of any type.

## Discussion

This study analysed variation in the use of investigations in general practices prior to cancer diagnosis by considering a wide range of patients and practice factors using a nationally representative sample of patients with cancer in England. The use of tests varied greatly among practices, indicating very different tendencies of investigation in different practices. This variation was not explained by patient characteristics; rather, if the same patients attended all practices, we would expect even larger variation, as patients with characteristics associated with greater test use were concentrated in practices with lower than average use of tests. Investigations are used more or less frequently in different patient groups, for example, patients with different cancers, but these patient group differences appear independent of practice variation. Higher levels of comorbidity were associated with lower use of investigations. This may reflect the fact that recent use of investigations, such as blood tests, is higher in this patient group, leading GPs to be less inclined to order new blood tests for new symptoms. However, this hypothesis needs to be substantiated by future research. While we see some small difference in test use by practices with different characteristics, most between-practice variation remained unexplained. Lower use of investigations in rural practices may reflect barriers to attending hospitals (eg, transport/travel) or differences in availability of direct access to investigations. Higher use of any type of tests in practices serving older populations may reflect more awareness of cancer due to more frequent exposure or a more morbid population requiring more frequent blood tests or investigations. It was not possible to analyse organisational variation for endoscopy use, reflecting the infrequent access to direct endoscopy by general practice.

### Comparison with literature

Prior work has examined variation in general practice regarding measures and markers of care quality, patient experience and diagnostic activity of relevance to cancer. The present study is reminiscent of previous work examining the component of practice variation that is due to patient case-mix; like observed previously, patient case-mix can both ‘obscure’ variation (as observed in our study) and also explain aspects of it.^21^ Other work has found practices rated higher for different aspects of patient experience have a greater/lower tendency for referrals or specialist investigations.^11^ However, we found no such association, although this may reflect a lack of power. In principle, some variation between practices may reflect chance, particularly in smaller practices and for rarer events (eg, imaging or endoscopy). However, our methods—specifically the use of random effects for practice in the regression models—account for such variation.^3^ Overall, our study aligns with previous findings while expanding the evidence on variation in primary care diagnostic activity for cancer by providing insights into a range of investigations and examining a series of hypotheses about likely mechanisms.

### Strengths and weaknesses

This study made use of nationally representative population-base data that contained information on a range of practice characteristics. Data are curated by clinicians ensuring high quality. Unlike many studies examining diagnostic processes in primary care, this study does not rely on the accuracy and completeness of recording of information, for example, regarding symptoms. However, this study does rely on clinicians auditing the diagnostic activity within their own practice, and as such different interpretation or engagement with the process may lead to some of the between-practice variation highlighted in this study. We have excluded data from 16% of patients due to missing data. However, as these missing data relate to the outcome of the regression models, the analyses are unbiased under the missing at random assumption, given the variables included in the analysis model. This is the same assumption that would apply to a multiple imputation approach. Given this, we have not imputed missing data for our outcome variables, as this would not lead to any reduction in bias, and may reduce the precision of estimates. While we aim for the findings to guide policy nationwide, it is important to recognise that only about 20% of all English general practices participated in the NCDA. Nonetheless, there was little difference in a range of characteristics between participating and non-participating practices.^16^ However, given the particular focus of the NCDA, it is unlikely that these findings can be generalised outside of the UK. While we have been able to examine and characterise general practice variation, two additional ‘levels’ of variation are of prior interest (that we were not able to investigate due to lack of data): within-practice variation between GPs; and between-practice variation within a broader structure such as the Clinical Commissioning Group or Integrated Care System. Prior work indicates that appreciable levels of between-GP variation exists in different aspects of prescribing activity or of patient experience, even within the same practice^12 22^; and then similarly broader health economy variation exists above and beyond that observed between practices.^23^ These research questions should be addressed by further work.

While in this study we have demonstrated substantial variation in investigation use between different healthcare providers and patient groups, we do not know what the optimum level of test investigation is. However, given the sizeable variation between general practices in investigation use, it follows that some practices may be overusing or underusing investigations, thereby providing suboptimal care. The lack of clarity surrounding the optimum level of test use may indeed be responsible, to some degree, for the observed variation. Determining the appropriate level of test use should be the focus of future research, acknowledging that levels may vary according to test type (the potential harms associated with a blood test are likely smaller than those associated with imaging or endoscopy) and patient characteristics. One approach is to examine measures of cancer stage at diagnosis or cancer survival at practice-level, but such approaches are limited by constrained reliability of practice-level measures of cancer outcomes, as cancer is a rare disease.^3^ Aggregation of activity over multiple years of data may increase reliability (reduce the influence of chance) but findings lack interpretability. Another approach is to examine other established measures of diagnostic care quality (such as rate of discordance with guideline-recommended urgent referrals or specialist investigations) at practice level, and aim to correlate them with level of test use. Further work is also required to investigate whether the use of tests in primary care leads to more or less timely cancer diagnoses.

## Conclusion

It is unclear from our data to what extent the observed large variation indicates the need for practices with lower test use to increase their test or vice versa. However, the large variation implies that there is potential to improve diagnostic pathways and the timeliness of cancer diagnosis by addressing unwarranted variation. Once optimal levels of testing are elucidated by other research, subsequent interventions should be designed at the practice level (depending on level of testing), as our findings indicate that it is the dominant course of variation. Rural practices may benefit from improved access to imaging and endoscopy, and practices serving younger populations may also be targeted for intervention. As with prescribing volume and other practice-level metrics, it will be beneficial for practices to receive regular updates on their relative use of investigations, such as blood tests, compared with other practices to support reflective practice.

## Supplementary material

10.1136/bmjqs-2024-017264 online supplemental file 1

## Data Availability

Data are available on reasonable request.

## References

[1] NHS NHS cancer overview. https://www.nhs.uk/conditions/cancer.

[2] Neal RD, Tharmanathan P, France B (2015). Is increased time to diagnosis and treatment in symptomatic cancer associated with poorer outcomes? Systematic review. Br J Cancer.

[3] Abel G, Saunders CL, Mendonca SC (2018). Variation and statistical reliability of publicly reported primary care diagnostic activity indicators for cancer: a cross-sectional ecological study of routine data. *BMJ Qual Saf*.

[4] Abel G, Elliott MN (2019). Identifying and quantifying variation between healthcare organisations and geographical regions: using mixed-effects models. BMJ Qual Saf.

[5] Bradley SH, Barclay M, Cornwell B (2022). Associations between general practice characteristics and chest X-ray rate: an observational study. Br J Gen Pract.

[6] Rubin GP, Saunders CL, Abel GA (2015). Impact of investigations in general practice on timeliness of referral for patients subsequently diagnosed with cancer: analysis of national primary care audit data. Br J Cancer.

[7] Cranfield BM, Koo MM, Abel GA (2023). Primary care blood tests before cancer diagnosis: National Cancer Diagnosis Audit data. Br J Gen Pract.

[8] Cranfield BM, Abel GA, Swann R (2023). Pre-Referral Primary Care Blood Tests and Symptom Presentation before Cancer Diagnosis: National Cancer Diagnosis Audit Data. Cancers (Basel).

[9] Swann R, McPhil S, Abel G (2023). Comparison between the 2018 and 2014 National Cancer Diagnosis Audits for England. Br J Gen Pract.

[10] Mendonca SC, Abel GA, Gildea C (2019). Associations between general practice characteristics with use of urgent referrals for suspected cancer and endoscopies: a cross-sectional ecological study. Fam Pract.

[11] Lyratzopoulos G, Mendonca SC, Gildea C (2018). Associations between diagnostic activity and measures of patient experience in primary care: a cross-sectional ecological study of English general practices. Br J Gen Pract.

[12] Guthrie B, Donnan PT, Murphy DJ (2015). Bad apples or spoiled barrels? Multilevel modelling analysis of variation in high-risk prescribing in Scotland between general practitioners and between the practices they work in. BMJ Open.

[13] Jäger L, Rosemann T, Burgstaller JM (2022). Quality and variation of care for chronic kidney disease in Swiss general practice: A retrospective database study. PLoS ONE.

[14] Sexton V, Atherton H, Dale J (2023). Clinician-led secondary triage in England’s urgent care delivery: a cross-sectional study. Br J Gen Pract.

[15] Roberts MJ, Campbell JL, Abel GA (2014). Understanding high and low patient experience scores in primary care: analysis of patients’ survey data for general practices and individual doctors. BMJ.

[16] Swann R, McPhail S, Abel GA (2023). National Cancer Diagnosis Audits for England 2018 versus 2014: a comparative analysis. Br J Gen Pract.

[17] Swann R, McPhail S, Witt J (2018). Diagnosing cancer in primary care: results from the National Cancer Diagnosis Audit. Br J Gen Pract.

[18] England N (2024). GP patient survey. https://gp-patient.co.uk.

[19] Office for Health Improvement and Disparities (2024). Cancer services. https://fingertips.phe.org.uk/profile/cancerservices.

[20] Office for Health Improvement and Disparities (2024). National general practice profiles. https://fingertips.phe.org.uk/profile/general-practice.

[21] Paddison C, Elliott M, Parker R (2012). Should measures of patient experience in primary care be adjusted for case mix? Evidence from the English General Practice Patient Survey. *BMJ Qual Saf*.

[22] Round T, Abel G (2020). Seeing the wood and the trees: the impact of the healthcare system on variation in primary care referrals. BMJ Qual Saf.

[23] Burton C, O’Neill L, Oliver P (2020). Contribution of primary care organisation and specialist care provider to variation in GP referrals for suspected cancer: ecological analysis of national data. *BMJ Qual Saf*.

