# Supplementary material for: Variation in the use of primary care-led investigations prior to a cancer diagnosis: analysis of the National Cancer Diagnosis Audit
Source: BMJ Qual Saf. 2024 Oct 23;34(6):e017264. doi: 10.1136/bmjqs-2024-017264 (PMC12171502; doi:10.1136/bmjqs-2024-017264)
Supplement: online supplemental file 1 [file bmjqs-34-6-s001.pdf]

Appendix 1

Table 1: Name and ICD 10 codes of the 29 cancer sites

Cancer Site	ICD-10 code		Cancer Site	ICD-10 code
Bladder	C67		Myeloma	C90
Brain	C71		Oesophageal	C15
Female Breast	C50		Oral/oropharyngeal	C1-C6, C9, C10
Cervical	C53		Ovarian	C56, C57
Colon	C18		Pancreatic	C25
Endrometrial	C54, C55		Prostate	C61
Gallbladder	C23		Rectal	C19-C20
Laryngeal	C32		Renal	C64
Leukaemia	C91-C95		Small intestine	C17
Liver	C22		Stomach	C16
Lung	C33, C34		Testicular	C62
Lymphoma	C81-C83, C85		Thyroid	C73
Melanoma	C43		Unknown primary	C77-C80
Mesothelioma	C45		--	--

Table 2: Adjusted odds ratios and 95% confidence intervals of the use of primary care led investigation among patients subsequently diagnosed with cancer (Model 2)

	Any Investigation			Blood Test			Imaging		
	OR (CI)	P-value	Global P-Value	OR (CI)	P-value	Global P-Value	OR (CI)	P-value	Global P-Value
**Gender**									
Male	Ref								
Female	1.04 (0.99, 1.09)	0.152		0.98 (0.94, 1.03)	0.539		1.08 (1.02, 1.14)	0.007	
**Ethnicity**									
White	Ref								
Other	0.94 (0.88, 1.01)	0.101		0.91 (0.85, 0.98)	0.012		0.97 (0.90, 1.05)	0.470	
**Age group**									
15-49	0.98 (0.90, 1.07)	0.654	0.001	0.91 (0.83, 0.99)	0.034	0.089	1.31 (1.19, 1.44)	<0.001	<0.001
50-59	0.98 (0.92, 1.05)	0.63		0.95 (0.88, 1.02)	0.129		1.03 (0.95, 1.12)	0.424	
60-69	Ref								
70-79	0.97 (0.92, 1.03)	0.274		1.01 (0.96, 1.07)	0.611		0.96 (0.90, 1.02)	0.195	
80+	0.88 (0.83, 0.93)	<0.001		1.00 (0.94, 1.06)	0.927		0.78 (0.73, 0.84)	<0.001	
**Deprivation**									
1-least deprived	Ref								
2	1.01 (0.95, 1.08)	0.666	0.253	1.04 (0.98, 1.11)	0.218	<0.001	0.98 (0.91, 1.06)	0.653	0.550
3	0.96 (0.90, 1.03)	0.259		0.93 (0.87, 0.99)	0.027		0.97 (0.90, 1.04)	0.371	
4	0.98 (0.91, 1.04)	0.453		0.93 (0.87, 1.00)	0.041		1.03 (0.95, 1.11)	0.464	
5-most deprived	0.95 (0.89, 1.01)	0.117		0.90 (0.85, 0.97)	0.002		0.99 (0.92, 1.06)	0.837	
**Morbidities**									
0	Ref								
1	0.86 (0.81, 0.92)	<0.001	<0.001	0.87 (0.82, 0.92)	<0.001	<0.001	0.93 (0.86, 0.99)	0.025	<0.001
2	0.79 (0.74, 0.85)	<0.001		0.83 (0.77, 0.88)	<0.001		0.87 (0.81, 0.94)	<0.001	
3	0.74 (0.69, 0.80)	<0.001		0.78 (0.72, 0.84)	<0.001		0.81 (0.74, 0.88)	<0.001	

4⁺	0.66 (0.61, 0.72)	<0.001		0.73 (0.68, 0.80)	<0.001		0.73 (0.66, 0.80)	<0.001	
**Cancer site**									
Bladder	1.04 (0.92, 1.19)	0.508	<0.001	0.46 (0.40, 0.52)	<0.001	<0.001	0.84 (0.70, 0.99)	0.039	<0.001
Brain	0.21 (0.17, 0.25)	<0.001		0.19 (0.16, 0.23)	<0.001		0.60 (0.47, 0.77)	<0.001	
Breast	0.03 (0.03, 0.04)	<0.001		0.03 (0.02, 0.03)	<0.001		0.13 (0.11, 0.16)	<0.001	
Cervical	0.45 (0.35, 0.57)	<0.001		0.19 (0.14, 0.26)	<0.001		0.96 (0.69, 1.34)	0.831	
Colon	Ref								
Endometrial	0.46 (0.40, 0.52)	<0.001		0.23 (0.20, 0.26)	<0.001		1.86 (1.61, 2.15)	<0.001	
Gallbladder	1.13 (0.80, 1.60)	0.481		0.87 (0.63, 1.21)	0.445		3.25 (2.32, 4.54)	<0.001	
Laryngeal	0.37 (0.30, 0.47)	<0.001		0.17 (0.13, 0.22)	<0.001		2.16 (1.68, 2.75)	<0.001	
Leukaemia	1.50 (1.30, 1.73)	<0.001		1.68 (1.46, 1.93)	<0.001		0.73 (0.61, 0.88)	0.001	
Liver	0.85 (0.73, 0.99)	0.043		0.74 (0.63, 0.86)	<0.001		2.71 (2.29, 3.21)	<0.001	
Lung	0.97 (0.89, 1.06)	0.518		0.32 (0.30, 0.35)	<0.001		6.07 (5.49, 6.70)	<0.001	
Lymphoma	0.88 (0.79, 0.98)	0.022		0.62 (0.56, 0.69)	<0.001		2.50 (2.21, 2.83)	<0.001	
Melanoma	0.05 (0.04, 0.06)	<0.001		0.02 (0.02, 0.02)	<0.001		0.08 (0.06, 0.11)	<0.001	
Mesothelioma	1.42 (1.14, 1.77)	0.002		0.39 (0.32, 0.48)	<0.001		10.12 (8.13, 12.60)	<0.001	
Myeloma	1.50 (1.27, 1.77)	<0.001		1.42 (1.21, 1.67)	<0.001		2.01 (1.69, 2.38)	<0.001	
Oesophageal	0.58 (0.51, 0.66)	<0.001		0.49 (0.43, 0.55)	<0.001		0.67 (0.55, 0.80)	<0.001	
Oral	0.19 (0.16, 0.22)	<0.001		0.15 (0.12, 0.17)	<0.001		0.83 (0.70, 0.99)	0.046	
Ovarian	1.18 (1.01, 1.37)	0.032		0.86 (0.74, 0.98)	0.021		3.89 (3.35, 4.52)	<0.001	
Pancreatic	1.22 (1.07, 1.38)	0.003		1.03 (0.91, 1.16)	0.602		2.50 (2.19, 2.87)	<0.001	
Prostate	3.28 (2.98, 3.61)	<0.001		3.34 (3.05, 3.66)	<0.001		0.45 (0.40, 0.51)	<0.001	
Rectal	0.76 (0.68, 0.85)	<0.001		0.80 (0.72, 0.90)	<0.001		0.34 (0.28, 0.42)	<0.001	
Renal	0.75 (0.66, 0.85)	<0.001		0.39 (0.34, 0.44)	<0.001		2.55 (2.22, 2.92)	<0.001	
Small intestine	0.95 (0.72, 1.25)	0.7		0.82 (0.63, 1.07)	0.188		1.38 (1.01, 1.88)	0.045	
Stomach	1.14 (0.98, 1.33)	0.094		0.94 (0.81, 1.09)	0.246		1.03 (0.85, 1.25)	0.748	
Testicular	0.78 (0.62, 0.98)	0.031		0.08 (0.06, 0.11)	<0.001		4.63 (3.68, 5.83)	<0.001	
Thyroid	1.05 (0.87, 1.27)	0.593		0.48 (0.40, 0.57)	<0.001		4.12 (3.41, 4.97)	<0.001	
Vulval	0.10 (0.07, 0.16)	<0.001		0.06 (0.04, 0.11)	<0.001		0.26 (0.14, 0.49)	<0.001	

Unknown primary	0.78 (0.68, 0.90)	0.001		0.60 (0.52, 0.69)	<0.001		2.48 (2.12, 2.90)	<0.001	
Other	0.47 (0.42, 0.52)	<0.001		0.33 (0.30, 0.37)	<0.001		1.45 (1.28, 1.65)	<0.001	

Table 3: Odds ratios and 95% confidence intervals in the mixed model (adjusted for patients and practice factors) of the use of investigation among patients subsequently diagnosed with cancer (Model 4)

	**Any Investigation**			**Blood Tests**			**Imaging**		
	**OR (CI)**	**P-value**	**Global P-Value**	**OR (CI)**	**P-value**	**Global P-Value**	**OR (CI)**	**P-value**	**Global P-Value**
**Gender**									
Male	Ref								
Female	1.03 (0.98, 1.08)	0.285		0.98 (0.93, 1.03)	0.483		1.07 (1.01, 1.13)	0.021	
**Ethnicity**									
White	Ref								
Non-white	0.97 (0.89, 1.05)	0.445		0.95 (0.88, 1.03)	0.251		0.98 (0.89, 1.06)	0.606	
**Age group**									
15-49	0.95 (0.87, 1.04)	0.293	0.002	0.88 (0.80, 0.97)	0.012	0.026	1.28 (1.16, 1.42)	<0.001	0.649
50-59	0.97 (0.90, 1.04)	0.397		0.95 (0.88, 1.02)	0.156		1.02 (0.94, 1.11)	0.619	
60-69	Ref								
70-79	0.97 (0.92, 1.03)	0.387		1.03 (0.97, 1.09)	0.384		0.97 (0.90, 1.03)	0.313	
80^{+}	0.88 (0.82, 0.94)	<0.001		0.99 (0.93, 1.06)	0.856		0.79 (0.73, 0.85)	<0.001	
**Deprivation**									
1-least deprived	Ref								
2	1.00 (0.93, 1.07)	0.942	0.267	1.02 (0.94, 1.10)	0.642	0.002	0.98 (0.90, 1.07)	0.656	0.350
3	0.96 (0.89, 1.03)	0.277		0.92 (0.85, 0.99)	0.023		0.97 (0.89, 1.05)	0.420	
4	0.97 (0.90, 1.05)	0.454		0.91 (0.84, 0.98)	0.019		1.04 (0.96, 1.33)	0.317	

5-most deprived	0.94 (0.86, 1.00)	0.051		0.89 (0.82, 0.96)	0.004		0.98 (0.90, 1.07)	0.637	
**Morbidities**									
0	Ref								
1	0.84 (0.79, 0.90)	<0.001	<0.001	0.86 (0.80, 0.91)	<0.001	<0.001	0.90 (0.84, 0.97)	0.005	<0.001
2	0.77 (0.72, 0.83)	<0.001		0.81 (0.75, 0.87)	<0.001		0.86 (0.80, 0.93)	<0.001	
3	0.72 (0.67, 0.78)	<0.001		0.77 (0.71, 0.84)	<0.001		0.80 (0.73, 0.88)	<0.001	
4^{+}	0.63 (0.58, 0.69)	<0.001		0.70 (0.64, 0.77)	<0.001		0.71 (0.64, 0.79)	<0.001	
**Cancer site**									
Bladder	1.02 (0.89, 1.17)	0.554	<0.001	0.43 (0.37, 0.49)	<0.001	<0.001	0.83 (0.70, 0.99)	0.049	<0.001
Brain	0.20 (0.16, 0.24)	<0.001		0.18 (0.15, 0.23)	<0.001		0.59 (0.45, 0.77)	<0.001	
Breast	0.03 (0.02, 0.03)	<0.001		0.02 (0.02, 0.03)	<0.001		0.13 (0.11, 0.16)	<0.001	
Cervical	0.46 (0.35, 0.60)	<0.001		0.19 (0.14, 0.25)	<0.001		1.03 (0.73, 1.46)	0.858	
Colon	Ref								
Endometrial	0.43 (0.38, 0.50)	0.489		0.21 (0.18, 0.25)	<0.001		1.83 (1.57, 2.13)	<0.001	
Gallbladder	1.13 (0.78, 1.64)	<0.001		0.90 (0.63, 1.27)	0.539		3.24 (2.26, 4.62)	<0.001	
Laryngeal	0.36 (0.28, 0.46)	<0.001		0.16 (0.12, 0.21)	<0.001		2.13 (1.64, 2.76)	<0.001	
Leukaemia	1.47 (1.26, 1.72)	0.029		1.66 (1.43, 1.93)	<0.001		0.69 (0.57, 0.84)	<0.001	
Liver	0.82 (0.69, 0.97)	0.371		0.70 (0.59, 0.83)	<0.001		2.74 (2.29, 3.29)	<0.001	
Lung	0.95 (0.87, 1.04)	0.015		0.30 (0.28, 0.33)	<0.001		6.22 (5.60, 6.92)	<0.001	
Lymphoma	0.87 (0.77, 0.98)	<0.001		0.59 (0.52, 0.66)	<0.001		2.54 (2.22, 2.89)	<0.001	
Melanoma	0.04 (0.04, 0.05)	0.003		0.02 (0.01, 0.02)	<0.001		0.08 (0.06, 0.11)	<0.001	
Mesothelioma	1.42 (1.12, 1.81)	<0.001		0.39 (0.31, 0.48)	<0.001		10.27 (8.13, 12.98)	<0.001	
Myeloma	1.47 (1.22, 1.76)	<0.001		1.39 (1.17, 1.66)	<0.001		2.05 (1.71, 2.46)	<0.001	
Oesophageal	0.56 (0.48, 0.64)	<0.001		0.45 (0.39, 0.52)	<0.001		0.64 (0.53, 0.79)	<0.001	
Oral	0.18 (0.15, 0.21)	0.035		0.14 (0.12, 0.17)	<0.001		0.83 (0.69, 1.01)	0.069	
Ovarian	1.15 (0.98, 1.35)	0.003		0.81 (0.70, 0.95)	0.008		3.81 (3.24, 4.48)	<0.001	
Pancreatic	1.17 (1.02, 1.35)	<0.001		1.00 (0.88, 1.14)	0.969		2.50 (2.16, 2.89)	<0.001	
Prostate	3.33 (3.01, 3.69)	<0.001		3.41 (3.09, 3.76)	<0.001		0.44 (0.39, 0.50)	<0.001	
Rectal	0.73 (0.64, 0.83)	<0.001		0.76 (0.68, 0.86)	<0.001		0.34 (0.27, 0.42)	<0.001	

Renal	0.74 (0.65, 0.84)	0.821		0.36 (0.32, 0.42)	<0.001		2.58 (2.23, 2.98)	<0.001	
Small intestine	0.96 (0.72, 1.29)	0.172		0.83 (0.63, 1.10)	0.203		1.22 (0.88, 1.72)	0.236	
Stomach	1.13 (0.95, 1.33)	0.033		0.91 (0.78, 1.07)	0.241		1.02 (0.84, 1.25)	0.847	
Testicular	0.78 (0.61, 1.00)	0.527		0.07 (0.05, 0.10)	<0.001		4.81 (3.76, 6.14)	<0.001	
Thyroid	1.06 (0.86, 1.30)	<0.001		0.48 (0.39, 0.58)	<0.001		4.35 (3.56, 5.32)	<0.001	
Vulval	0.10 (0.07, 0.16)	<0.001		0.06 (0.03, 0.10)	<0.001		0.29 (0.15, 0.56)	<0.001	
Unknown primary	0.79 (0.67, 0.92)	<0.001		0.60 (0.51, 0.70)	<0.001		2.59 (2.20, 3.06)	<0.001	
Other	0.45 (0.40, 0.50)	0.489		0.31 (0.27, 0.35)	<0.001		1.46 (1.27, 1.67)	<0.001	
**Practice level factors**									
**Practice Deprivation**									
1-least deprived	Ref								
2	1.03 (0.93, 1.15)	0.552	0.295	1.08 (0.96, 1.20)	0.19	0.29	0.94 (0.85, 1.04)	0.257	0.819
3	1.08 (0.97, 1.21)	0.144		1.11 (1.00, 1.24)	0.061		0.97 (0.88, 1.07)	0.588	
4	1.03 (0.92, 1.15)	0.609		1.11 (0.99, 1.24)	0.069		0.96 (0.86, 1.06)	0.434	
5-most deprived	1.04 (0.93, 1.17)	0.502		1.13 (1.00, 1.27)	0.051		0.94 (0.85, 1.05)	0.355	
**Rurality**									
Urban practice	Ref								
Rural practice	0.86 (0.76, 0.96)	0.010		0.90 (0.80, 1.02)	0.105		0.91 (0.81, 1.02)	0.088	
**Total patients per GP**									
Size 1	Ref								
Size 2	1.06 (0.98, 1.15)	0.167	0.162	1.11 (1.02, 1.21)	0.016	0.024	1.02 (0.95, 1.11)	0.537	0.819
Size 3	1.08 (0.97, 1.19)	0.158		1.14 (1.03, 1.27)	0.013		1.01 (0.92, 1.12)	0.790	
GPPS Access	1.01 (0.95, 1.08)	0.641		1.01 (0.95, 1.07)	0.791		0.97 (0.92, 1.03)	0.397	
GPPS Continuity	1.01 (0.96, 1.07)	0.654		1.01 (0.96, 1.07)	0.709		1.01 (0.96, 1.05)	0.785	
GPPS Satisfaction	0.98 (0.89, 1.08)	0.656		1.00 (0.90, 1.10)	0.975		1.07 (0.98, 1.17)	0.130	
GPPS doctors Communication	1.03 (0.97, 1.11)	0.337		1.05 (0.98, 1.13)	0.175		0.95 (0.89, 1.01)	0.123	
TWW referral	1.02 (0.98, 1.05)	0.345		1.00 (0.96, 1.03)	0.783		1.04 (1.00, 1.07)	0.030	
MaxQoF	0.97 (0.93, 1.01)	0.094		0.98 (0.94, 1.01)	0.193		0.99 (0.97, 1.03)	0.907	

Patients per GP	1.02 (0.98, 1.05)	0.339		1.03 (0.99, 1.07)	0.102		0.98 (0.95, 1.01)	0.227	
Patients over 65 per GP	1.05 (1.01, 1.10)	0.016		1.04 (1.00, 1.09)	0.058		1.04 (1.00, 1.08)	0.046	

Table 4: Characteristics of primary care patients subsequently diagnosed with cancer based on the use of investigation

Exposure variables	Total Patients N=53,252 (column %)	Any test N=29,932 (row %)	Blood Tests N=23,422 (row %)	Imaging N= 12,368 (row %)	Endoscopy N= 845 (row %)
**Gender**					
Male	29,155 (54.8)	19,224 (66.0)	15,867 (54.4)	6,441 (22.1)	474 (1.6)
Female	24,097 (45.3)	10,708 (44.4)	7,555 (31.4)	5,927 (24.6)	371 (1.5)
**Ethnicity**					
White	46,297 (90.8)	26,029 (56.2)	20,322 (44.0)	10,822 (23.4)	729 (1.6)
Other	4,686 (9.2)	2,575 (55.0)	1,993 (42.5)	1,074 (23.0)	89 (1.9)
**Age group**					
15-49	5,610 (10.6)	2,313 (41.2)	1,510 (26.9)	1,412 (25.2)	79 (1.4)
50-59	7,421 (14.0)	3,961 (53.4)	3,024 (40.8)	1,675 (22.6)	114 (1.5)
60-69	12,385 (23.4)	7,592 (61.3)	6,056 (49.0)	2,915 (23.5)	209 (1.7)
70-79	15,874 (30.0)	9,606 (60.5)	7,643 (48.2)	3,873 (24.4)	262 (1.6)
80^{+}	11,711 (22.1)	6,390 (54.6)	5,139 (44.0)	2,469 (21.1)	181 (1.5)
**Deprivation**					
1 – least deprived	10,792 (20.3)	6,058 (56.1)	4,952 (46.0)	2,212 (20.5)	156 (1.5)
2	10,587 (20.0)	6,030 (57.0)	4,911 (46.4)	2,301 (21.7)	157 (1.5)
3	10,562 (19.8)	5,918 (56.0)	4,628 (43.8)	2,368 (22.4)	164 (1.6)
4	10,367 (19.5)	5,838 (56.3)	4,463 (43.1)	2,589 (25.0)	181 (1.7)
5 – most deprived	10,944 (20.6)	6,088 (55.6)	4,468 (40.8)	2,898 (26.5)	187 (1.7)
**Morbidities**					
0	12,301 (23.1)	6,589 (53.6)	5,106 (41.5)	2,793 (22.7)	187 (1.5)
1	15,948 (30.0)	9,247 (58.0)	7,300 (45.8)	3,685 (23.1)	240 (1.5)
2	12,539 (23.6)	7,258 (58.0)	5,704 (45.5)	3,000 (24.0)	190 (1.5)
3	7,239 (13.6)	4,074 (56.3)	3,178 (44.0)	1,696 (23.4)	130 (1.8)
4^{+}	5,225 (9.8)	2,764 (53.0)	2,134 (40.8)	1,194 (22.8)	98 (1.8)
**Cancer site**					
Bladder	1,487 (2.8)	977 (65.7)	603 (40.6)	215 (14.5)	10 (0.7)
Brain	699 (1.3)	202 (29.0)	154 (22.0)	83 (11.8)	1 (0.1)
Breast	5,881 (11.0)	380 (6.5)	244 (4.2)	190 (3.2)	5 (0.1)
Cervical	251 (0.5)	118 (47.0)	64 (25.5)	52 (20.7)	8 (3.2)
Colon	3,758 (7.1)	2,518 (65.1)	2,289 (61.0)	661 (17.6)	153 (4.1)
Endometrial	1,517 (3.0)	723 (47.7)	386 (25.4)	466 (30.7)	6 (0.4)
Gallbladder	157 (0.3)	106 (67.5)	87 (55.4)	63 (40.1)	4 (2.6)
Laryngeal	345 (0.7)	143 (41.5)	69 (20.0)	109 (31.6)	0 (0.0)
Leukaemia	1,399 (2.6)	1,004 (71.8)	971 (69.4)	185 (13.2)	6 (0.4)
Liver	832 (1.6)	502 (60.3)	425 (51.1)	301 (36.2)	18 (2.2)
Lung	6,960 (13.1)	4,461 (64.1)	2,238 (32.2)	3,879 (55.7)	70 (1.0)
Lymphoma	2,251 (4.2)	1,415 (62.9)	1,086 (48.3)	809 (36.0)	28 (1.2)
Melanoma	2,614 (5.0)	228 (8.7)	75 (3.0)	49 (1.8)	0 (0.0)
Mesothelioma	450 (1.0)	328 (72.9)	169 (37.6)	304 (67.6)	1 (0.2)
Myeloma	867 (1.6)	642 (74.1)	591 (68.2)	257 (29.6)	11 (1.3)
Oesophageal	1,368 (2.6)	715 (52.3)	575 (42.0)	170 (12.4)	153 (11.2)
Oral	1,118 (2.1)	308 (27.6)	203 (18.2)	181 (16.2)	3 (0.3)

Ovarian	1,152 (2.2)	814 (70.7)	645 (56.0)	565 (49.1)	19 (1.7)
Pancreatic	1,623 (3.1)	1,128 (69.5)	985 (60.7)	560 (34.5)	64 (4.0)
Prostate	9,119 (17.1)	7,863 (86.2)	7,630 (83.7)	792 (8.7)	20 (0.2)
Rectal	1,888 (3.6)	1,146 (60.7)	1,062 (54.5)	131 (6.9)	87 (4.6)
Renal	1,585 (3.0)	930 (58.7)	580 (36.6)	574 (36.2)	20 (1.3)
Small intestine	248 (0.5)	159 (64.1)	136 (54.8)	58 (23.4)	15 (6.1)
Stomach	972 (1.8)	662 (68.1)	564 (58.0)	175 (18.0)	106 (11.0)
Testicular	422 (0.8)	265 (62.8)	43 (10.2)	244 (57.8)	0 (0.0)
Thyroid	638 (1.2)	431 (67.6)	260 (40.8)	330 (51.7)	0 (0.0)
Vulval	181 (0.3)	30 (16.6)	15 (8.3)	10 (5.5)	1 (0.6)
Unknown primary	1,048 (2.0)	621 (59.3)	494 (47.1)	355 (33.8)	15 (1.4)
Other	2,422 (4.6)	1,141 (47.1)	786 (32.5)	600 (24.8)	21 (0.8)

Table 5: Between-practice variation in the adjusted mixed models by varying patient factors

Variables	Any investigation SD (95% CI)	Blood test SD (95% CI)	Imaging SD (95% CI)
Age, gender, ethnicity	0.37 (0.34-0.40)	0.38 (0.36-0.41)	0.29 (0.26-0.32)
Age, gender, ethnicity, deprivation	0.37 (0.34-0.40)	0.38 (0.36-0.41)	0.27 (0.24-0.31)
Age, gender, ethnicity, deprivation, comorbidity	0.37 (0.34-0.40)	0.39 (0.36-0.42)	0.27 (0.24-0.31)
